# Special Issue “Molecular Advances in Biomarkers of Central Nervous System Diseases and Disorders”

**DOI:** 10.3390/ijms27177941

**Published:** 2026-09-06

**Authors:** Chiara Villa, David Gurwitz

**Affiliations:** 1 School of Medicine and Surgery, University of Milano-Bicocca, 20900 Monza, Italy; 2 Department of Molecular Genetics and Computational Medicine, Faculty of Health and Medical Sciences, Tel Aviv University, Tel Aviv 69978, Israel; gurwitz@tauex.tau.ac.il

Central nervous system (CNS) diseases and disorders have posed a substantial challenge to global health, contributing to disability, mortality, and healthcare costs [1,2]. These conditions impose a significant burden on individuals, families, and society worldwide, frequently resulting in severe impairments in motor and cognitive abilities that limit patients’ autonomy and quality of life [3,4]. Given the increasing prevalence and complex pathophysiology of these diseases and disorders, there is an urgent need for improved diagnostic and prognostic strategies [5,6]. In this context, molecular biomarkers have emerged as valuable tools for early detection, prognostic assessment, patient stratification, and the development of tailored patient-specific treatment options [7,8]. Recent advancements in multi-omics technologies integrating genomics, transcriptomics, epigenomics, proteomics, and metabolomics have significantly improved the identification of novel disease-specific biomarkers in a wide range of CNS disorders [9,10,11,12,13,14,15,16,17,18,19,20].

This Special Issue gathers five reviews and four original research articles that provide novel insights on the advancements of molecular biomarkers across different CNS conditions. As widely recognized, the pathological processes may start several years before a clear clinical diagnosis, underlying the crucial need for tools capable of identifying early molecular alterations predictive of the disease or disorder manifestation [21,22,23]. In this regard, the first review published in this Special Issue investigated the most relevant signaling molecules associated with cognitive impairment, Alzheimer’s disease (AD), and vascular dementia (VD) [24]. Through extensive panels of molecular biomarkers, researchers determined that progressive changes in the expression of specific signaling molecules may reflect different neurodegenerative and vascular-associated biological processes [24]. Recent studies have suggested additional biomarkers and directions for AD diagnosis and prognosis [25,26,27,28,29,30,31,32,33,34].

Cognitive impairment has been also investigated in schizophrenia; the authors of a review article in this Special Issue addressed the crucial role of protein phosphatase in an intracellular signaling pathway underlying synaptic dysfunction. Moreover, its potential as a novel therapeutic strategy and the limitations of current pharmacological approaches were examined [35].

The importance of a multimodal biomarker approach is further discussed by another review in this Special Issue linking mild behavioral impairment (MBI) to tauopathies, in which late-life behavioral symptoms may constitute an early clinical manifestation of tauopathy-mediated neurodegeneration [36], a pathogenic pathway suggested decades ago [37]. When combined with blood-based biomarkers, genotype-based stratification, and neuroimaging, MBI has the potential to improve early detection, differential diagnosis, and risk estimation [36].

Another article included in this Special Issue proposed the use of extracellular vesicles (EVs) as an emerging class of liquid biopsy biomarkers for spinal cord injury (SCI), a severe neurological condition characterized by heterogeneous injury mechanisms and limited tools for reliable early identification [38]. The relevance of CNS-enriched EVs for different brain disorders has been featured in recent systematic reviews and diagnostic meta-analyses [39,40,41,42,43,44]. Increasing evidence suggests that EV-based protein and microRNAs (miRNAs) reflect pathological processes in the CNS and are readily detectable in accessible biofluids, making them intriguing tools for refining diagnosis and prognosis in combination with a neurological examination and conventional imaging methods [38,45,46]. Concerning SCI research, another notable review in the current Special Issue highlighted the use of human spinal cord organoids-on-a-chip as a promising tool for modeling, drug screening, and regenerative therapy development. Compared to conventional 2D cultures and animal models, organoid-based platforms better resemble human spinal cord physiology and pathology, allowing novel insights into tailored therapies for SCI patients and accelerating the translation of preclinical findings into clinical applications [47,48].

In the novel research articles, two contributions evaluated the use of molecular biomarkers for multiple sclerosis (MS) in clinical practice. A multicenter study conducted in Central-Eastern European countries found considerable variability in the implementation of molecular biomarkers, especially in the testing of neurofilament light chain (NfL) and kappa free light chain (κFLC) proteins [49]. NfL and κFLC have recently emerged as promising biomarkers found both in the serum and in the cerebrospinal fluid of MS patients [50,51,52,53,54,55]. Apart from the utility of oligoclonal bands in the CSF, the authors of the study published in this Special Issue highlighted the urgent need for a standardized international consensus for MS biomarkers [56]. In line with this perspective, a longitudinal study evaluated the potential of serum NfL and glial fibrillary acidic protein (GFAP) as blood-based biomarkers for monitoring the therapeutic response of MS patients to dimethyl fumarate and ocrelizumab. The different dynamics observed under these treatments highlighted distinct pathological processes reflecting the two biomarkers [49].

Another study demonstrated the importance of combing whole-exome sequencing and cellular functional assays for identifying novel genes and pathways in early-onset forms of Parkinson’s disease (PD) [57,58,59]. In particular, the authors found that rare variants in lysosomal-related genes may impair autophagic and lysosomal homeostasis, thereby providing new insights into disease pathogenesis and paving the way for enhancing diagnosis, prognosis, and future therapeutic strategies [58].

A similar mechanistic perspective was provided by the experimental study investigating early morphological and molecular alterations induced by ischemia–reperfusion brain injury following cardiac arrest. Although further validation is required, the authors report for the first time a link between the increased expression of prolyl hydroxylase domain-1 (PHD-1) and severe metabolic abnormalities and impaired brain perfusion in a rat model for ischemia–reperfusion brain injury. These data suggested a key role for oxygen-sensing pathways in the pathogenesis of post-resuscitation brain injury and suggested PHD-1 as a potential therapeutic target for improving neurologic outcomes [60].

Collectively, these contributions provide an overview of the significance of molecular biomarkers in basic research, clinical diagnosis, prognosis and therapeutic options across several CNS disorders. The review and original research articles in this Special Issue integrate findings from genomics, transcriptomics, proteomics, EVs, and advanced experimental models. Continued research in this critical area holds great promise for the development of more effective diagnostic tools and, eventually, disease-modifying therapies that can improve the lives of individuals with these complex conditions.

## Abbreviations

The following abbreviations are used in this manuscript:

AD	Alzheimer’s disease
CNS	central nervous system
CSF	cerebrospinal fluid
EV	extracellular vesicle
κFLC	kappa free light chain
GFAP	glial fibrillary acidic protein
MBI	mild behavioral impairment
miRNA	microRNA
MS	multiple sclerosis
NfL	neurofilament light chain
PD	Parkinson’s disease
PHD-1	prolyl hydroxylase domain-1
SCI	spinal cord injury
VD	vascular dementia
